# Standardized Extract (HemoHIM) Protects against Scopolamine-Induced Amnesia in a Murine Model

**DOI:** 10.1155/2021/8884243

**Published:** 2021-03-17

**Authors:** Seul-Ki Kim, Da-Ae Kwon, Yong Sang Kim, Hak Sung Lee, Hyun Kyu Kim, Won-Ki Kim

**Affiliations:** ^1^Department of Neuroscience, Korea University College of Medicine, 145 Anam-ro, Seongbuk-gu, Seoul 02841, Republic of Korea; ^2^Efficacy Evaluation Team, Food Science R&D Center, Kolmar BNH Co., Ltd., 61 Heolleung-ro 8-gil, Seocho-gu, Seoul, Republic of Korea; ^3^Food Safety, Food Science R&D Center, Kolmar BNH Co., Ltd., 22-15 Sandan-gil, Jeonui-myeon, Sejong 30003, Republic of Korea; ^4^Natural Product Research Team, Food Science R&D Center, Kolmar BNH Co., Ltd., 61 Heolleung-ro 8-gil, Seocho-gu, Seoul, Republic of Korea; ^5^Food Science R&D Center, Kolmar BNH Co., Ltd., 61 Heolleung-ro 8-gil, Seocho-gu, Seoul, Republic of Korea

## Abstract

HemoHIM is a medicinal herbal preparation of *Angelica gigas* Nakai (Apiaceae), *Cnidium officinale* Makino (Umbelliferae), and *Paeonia lactiflora* Pallas (Paeoniaceae) designed for immune regulation. In the present study, the memory-enhancing effects of a standardized extract (HemoHIM) on scopolamine-induced memory impairment in a murine model was investigated. To induce amnesia, scopolamine (1 mg/kg) was intraperitoneally (i.p.) injected into mice 30 min before the start of behavioral tests. The Y-maze, novel object recognition test (NORT), and passive avoidance task (PAT) were used to evoke memory functions. HemoHIM significantly improved scopolamine-induced memory impairment in ICR mice, which was evidenced by an improvement of spontaneous alternation in the Y-maze, recognition index in NORT, and latency time in PAT. To elucidate the possible mechanism, the cholinergic activity and mRNA levels of choline acetyltransferase (ChAT), muscarinic acetylcholine receptor (mAchR), brain-derived neurotrophic factor (BDNF), and cAMP response element-binding protein (CREB) were measured using reverse transcription (RT-PCR) and western blot analyses, respectively. HemoHIM treatment attenuated the scopolamine-induced hyperactivation of acetylcholinesterase (AchE) activity. In addition, ChAT, mAchR, and CREB mRNA levels were increased in the hippocampus compared with the scopolamine group. Furthermore, HemoHIM treatment resulted in elevated BDNF protein expression. These results indicate that HemoHIM may exert antiamnesic activity by increasing Ach and inhibiting AchE in the hippocampus. In addition, HemoHIM has therapeutic potential by upregulating ChAT, mAchR, and BDNF, which is apparently mediated by activation of the CREB and ERK signaling pathways.

## 1. Introduction

The issue of aging is one of the most significant problems globally. Accordingly, the main problem is that social and economic costs associated with aging are increasing as the number of patients with degenerative diseases is rapidly increasing in the growing elderly population [1]. In addition, memory loss and amnesia have become important topics across all age groups in modern society. Progressive memory loss can cause serious social problems in degenerative diseases such as epilepsy, Alzheimer's disease, vascular dementia, as well as neurodegenerative and vascular disorders [2, 3].

The hippocampus and cortex of the brain are involved in maintaining and controlling memory. Memory loss and its severity are regulated by the neurotransmitter acetylcholine (Ach), which is a chemical messenger released by nerve cells to send signals to other cells, such as neurons, muscle cells, and gland cells [4]. Choline uptake and Ach synthesis are decreased in the hippocampus and cerebral cortex. Conversely, the expression of acetylcholinesterase (AchE), an enzyme that degrades Ach, is activated in dementia patients [5]. Therefore, AchE inhibitors such as tacrine, donepezil, and rivastigmine have been used as therapeutic agents. However, these drugs have short half-lives and side effects, including nausea and sleep disorders [6]. Conversely, natural materials and herbs exhibit fewer adverse effects and a mixture of various ingredients has the advantage of effectively controlling complex symptoms.

Recently, various health foods using food materials and natural products have been developed around the world, and the increase in consumer demand does not match the availability of pharmaceutical products; however, immunity [7, 8], liver activity [9], blood circulation improvement [10], and memory improvement [11, 12] are areas of current public interest.

The standardized extract HemoHIM is an herbal preparation consisting of roots of *Angelica gigas* Nakai (Apiaceae), *Cnidium officinale* Makino (Umbelliferae), and *Paeonia japonica* Miyabe (Paeoniaceae), which reportedly inhibits various activities of human mast cells, exerts anti-inflammatory effects on cigarette smoke and lipopolysaccharides, and ameliorates oxidative stress, such as stress induced by irradiation and immune-modulating activities [13–16]. HemoHIM is not a simple extract of Angelica Radix, Cnidium Rhizoma, and Paeonia Radix of Samul-Tang in oriental medicine, but rather a standardized extract of a polysaccharide fraction mixed in a certain proportion. The major components are gallic acid, chlorogenic acid, paeoniflorin, and nodakenin. We recently reported that the quantities of nodakenin, chlorogenic acid, and paeoniflorin were 106, 37, and 358 mg/100 g, respectively, using HPLC, and we used same batch of HemoHIM in this study as well [17]. In previous studies, the herbal ingredients of HemoHIM, or their compounds, showed protective effects against amnesia and dementia [18, 19]. In particular, Angelica Radix reportedly has preventive and therapeutic effects on brain function, such as improving cognitive memory and forgetfulness, and protects against beta amyloid-induced cytotoxicity [20]. Chlorogenic acid is effective in cognitive function by increasing Ach synthesis [21–23]. With the chlorogenic acid, HemoHIM is expected to have a positive effect on cognitive enhancement.

Scopolamine is a well-known anticholinergic drug and acts by blocking the muscarinic Ach receptor (mAchR) that can interfere with memory deficits by disrupting cholinergic neurotransmission [24]. In several studies, scopolamine was reportedly used to induce amnesia in experimental models of Alzheimer's disease and has been used to screen for antiamnesic drugs [25–27]. In the present study, the memory-enhancing effects of HemoHIM in a scopolamine-induced murine model was investigated using behavioral tests, i.e., the novel object recognition test (NORT), Y-maze, and passive avoidance task (PAT). The Ach and AchE levels were subsequently investigated in the hippocampus. In addition, brain-derived neurotrophic factor (BDNF), choline acetyltransferase (ChAT), mAchRs M1 and M2, and cAMP response element-binding protein (CREB) levels were measured using reverse transcription polymerase chain reaction (RT-PCR) analysis.

## 2. Materials and Methods

### 2.1. Materials

Donepezil hydrochloride monohydrate and scopolamine hydrobromide were purchased from Sigma Chemical Co. (St. Louis, MO, USA). Acetylcholine ELISA kit and acetylcholinesterase ELISA kit were purchased from Elabscience Biotechnology Co. (Houston, TX, USA). Anti-*β*-actin antibody was purchased from Sigma Chemical Co. Anti-BDNF antibodies were purchased from Abcam (Cambridge, UK). All other materials were obtained from normal commercial sources and were of the highest grade available.

### 2.2. Preparation of HemoHIM

HemoHIM was prepared as described in our previous report [14]. The batch (HHH009) of standardized HemoHIM containing chlorogenic acid (25–60 mg/100 g), paeoniflorin (200–400 mg/100 g), and nodakenin (50–150 mg/100 g) was manufactured by Kolmar BNH Co., Ltd. (Sejong Si, Republic of Korea). Briefly, the traditional Korean medicinal plants, Angelica Radix (root of *Angelica gigas* Nakai (Apiaceae)), Cnidii Rhizoma (rhizome of *Cnidium officinale* Makino (Umbelliferae)), and Paeonia Radix (root of *Paeonia lactiflora* Pallas (Paeoniaceae)), were extracted for 4 h in boiling water to obtain a total extract. Half of the extract was precipitated with 95% ethanol to obtain an ethanol-insoluble polysaccharide fraction. Finally, the HemoHIM adding polysaccharide fraction was concentrated to a solid content of 30 ± 3%, freeze-dried (MCFD8512; Ilshin Lab Co., Ltd., Seoul, Republic of Korea), and stored at 4°C until use.

### 2.3. Animal

Five-week-old male ICR mice were purchased from Doo Yeol Biotech (Seoul, Korea). Animals were housed in a temperature- and humidity-controlled room at 25 ± 2°C and 50 ± 20% relative humidity, with 12-h light/12-h dark cycles. All animals had free access to food and water. All animal experiments were approved by the Korea Kolmar Animal Experimental Ethics Committee and were performed in accordance with established regulations (Approval number: 18-KBH-S-01). Animals were randomly put into five different groups (*n* = 13). Treatment administration in the five groups was as follows:

  Group 1: normal saline, i.p. and p.o. (control)
  Group 2: scopolamine 1 mg/kg i.p. and normal saline p.o. (negative control)
   Group 3: scopolamine 1 mg/kg i.p. and donepezil 1 mg/kg p.o. (positive control)
   Group 4: scopolamine 1 mg/kg i.p. and HemoHIM 250 mg/kg p.o.
   Group 5: scopolamine 1 mg/kg i.p. and HemoHIM 500 mg/kg p.o.

**Figure 1:**
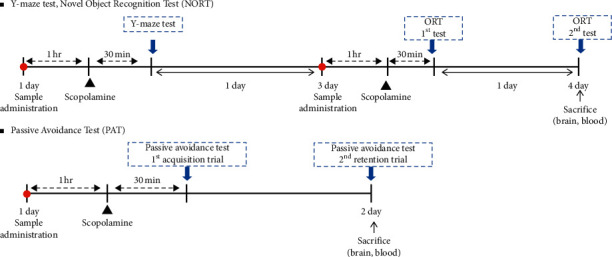
Schematic description of the behavioral experimental design. ICR mice were orally treated with vehicle, HemoHIM 250, 500 mg/kg, or donepezil 1 mg/kg before the behavioral experiment. Animals were randomly put into five different groups (*n* = 13). Scopolamine 1 mg/kg was i.p. injected 1 h after sample administration. The behavioral test was performed 30 min after scopolamine injection.

The volume of oral (p.o.) and intraperitoneal (i.p.) administrations was 10 mL/kg body weight of mice. For each behavioral test performed in this study, the mice were orally administered HemoHIM (250 or 500 mg/kg) or donepezil (1 mg/kg) as a positive control 90 min before the behavioral trial (Figure 1). Memory impairment was induced by intraperitoneal (i.p.) injection of scopolamine (1 mg/kg) 30 min after the administration of HemoHIM or donepezil. The control group received normal saline.

### 2.4. Y-Maze Task

The Y-maze task is used as a measure of immediate spatial working memory, a form of short-term memory [28]. The Y-maze is a three-arm horizontal maze (40 cm long and 3 cm wide with walls 12 cm high) in which the three arms are symmetrically separated at 120°. The apparatus floor and walls were constructed from black polyvinyl plastic. Mice were initially placed in one arm, and the arm entry sequence (e.g., ABCCAB, where letters indicate arm-codes) and the number of arm entries were recorded manually for each mouse over an 8-min period. An actual alternation was defined as entries into all three arms consecutively (i.e., ABC, CAB, or BCA but not BAB). Mice were orally administered HemoHIM (250 or 500 mg/kg) or donepezil (1 mg/kg) 1.5 h before the acquisition trial. Memory impairment was induced by scopolamine treatment (1 mg/kg, i.p.) 30 min after the administration of HemoHIM, donepezil, or normal saline. Maze arms were thoroughly cleaned between tests to remove residual odors. Percentage alternation was determined by dividing the total number of alternations by the total number of choices minus 2 multiplied by 100 as shown by the following equation:

Alternation (%) = (Number of alternations/Total arm entries − 2) × 100. The number of arm entries served as an indicator of locomotor activity. The number of total arm entries was measured as an indicator for animal behavior activity.

### 2.5. Novel Object Recognition Test

To assess an object recognition memory, the NORT was performed as previously described [29]. The NORT test was conducted 1 day after the Y-maze test. Mice were orally administered HemoHIM (250 or 500 mg/kg) or donepezil (1 mg/kg) 1½ *h* before NORT. Memory impairment was induced by scopolamine treatment (1 mg/kg, i.p.) 30 min after administration of HemoHIM or donepezil. The experimental apparatus consisted of a polyvinyl plastic square open field (25 cm × 25 cm × 25 cm). Habituation training was conducted by exposing the animal to the experimental apparatus for 5 min per day in the absence of objects. During the test, mice were placed in the experimental apparatus in the presence of two identical objects and allowed to explore for 5 min. After a retention interval of 24 h, mice were again placed in the apparatus; however, one of the objects was replaced with a novel object. Mice were also allowed to explore for 5 min. The objects chosen for this experiment were approximately of the same height. The duration of time mice spent exploring each object (the number of exploring familiar object, N1; the number of exploring the novel object, N2) was recorded. The recognition index (%) = N2/(N2 + N1) × 100.

### 2.6. Passive Avoidance Task

The PAT was conducted in identical illuminated and nonilluminated boxes (Avoidance System, version 1.1; BS Techno-lab Inc., Seoul, Korea). The test is an automatic learning method designed for active and passive avoidance experiments in animals. The test station consists of two chambers, and the bottom consists of a grid that can cause an electric shock. These two compartments were separated by a guillotine door (5 × 5 cm). For the acquisition trial, mice were initially placed in the illuminated compartment and the door between the two compartments was opened 10 sec later. When mice entered the dark compartment, the door automatically closed and an electrical foot shock (0.3 mA) 3 sec in duration was delivered through the stainless steel rods. Mice were again placed in the illuminated compartment 24 h after the acquisition trial to test retention. The time measured for a mouse to enter the dark compartment after the door opened was defined as latency up to 300 sec. The test was conducted as previously described with modifications [30].

### 2.7. RNA Isolation and RT-PCR

Total RNA was extracted from the hippocampus of ICR mice using the RNeasy Mini Kit (Qiagen, Hilden, Germany) and quantified with a NanoDrop 2000 UV-Vis spectrophotometer (Thermo Fisher Scientific Inc., Waltham, MA, USA). cDNA was synthesized from extracted total RNA using a High-Capacity cDNA Reverse Transcription Kit (Applied Biosystems, Carlsbad, CA, USA). The cDNA of genes of interest was amplified using AccuPower PCR Premix (Bioneer, Daejeon, Korea).  Table 1 shows the primer sequences used for RT-PCR. The amplified cDNA was electrophoresed on 1.8% agarose gel and stained with ethidium bromide (EtBr). The expression level of the target mRNA was measured using *β*-actin as a control and analyzed with Image J software (NIH, Framingham, MA, USA).

### 2.8. Biochemical Assay

Briefly, mice were sacrificed by decapitation and the brain was immediately removed to isolate the cortex or hippocampus. A hippocampus tissue was added to a microfuge tube containing cold phosphate-buffered saline (PBS) and homogenized using a homogenizer. The resulting tissue was then centrifuged at 13,000 rpm and 4°C. AchE and Ach levels in the hippocampus were determined using the acetylcholine ELISA kit and acetylcholinesterase ELISA kit (Elabscience Biotechnology Co.) according to the manufacturer's instructions.

### 2.9. Western Blotting

Isolated tissues were homogenized in ice-chilled Tris-HCl buffer (20 mM, pH 7.4). Homogenate samples (15 *μ*g of protein) were subjected to SDS-PAGE under reducing conditions. Proteins were transferred onto 20 *μ*m PVDF membranes in transfer buffer and further separated at 100 V for 2 h at 4°C to determine expression levels. The western blots were incubated for 2 h with blocking solution (5% skim milk) at 4°C followed by overnight incubation with primary antibodies. The blots were then washed twice with Tween 20/Tris-buffered saline (TTBS), incubated with a 1 : 5,000 dilution of horseradish peroxidase-conjugated secondary antibody for 1 h at room temperature, washed three times with TTBS, and developed using enhanced chemiluminescence (Amersham Life Science, Arlington Heights, IL, USA). Immunoblots were imaged using the bioimaging program on a LAS-4000 mini imager (Fujifilm Lifescience USA, Stamford, CT, USA) and analyzed using Multi Gauge version 3.2 (Fujifilm Holdings Corporation, Tokyo, Japan). The BDNF level was determined by calculating the ratios of BDNF to the corresponding *β*-actin on the same membranes.

### 2.10. Statistical Analysis

All results are presented as the mean ± standard error of the mean (S.E.M). One-way analysis of variance (ANOVA) followed by Duncan's multiple range test were used for comparing three or more groups. The GraphPad Prism5.0 (GraphPad Prism Software Inc., San Diego, CA, USA) program was used for statistical analysis. *P* values < 0.05 were considered statistically significant.

## 3. Results

### 3.1. Effects of HemoHIM on Learning and Memory Improvement in Behavioral Tests

All experimental groups showed normal results regarding general behavioral changes, and adverse events were not observed. In addition, body weight and food intake measured during treatment in all animal groups were not significantly different among the groups (data not shown).

**Figure 2:**
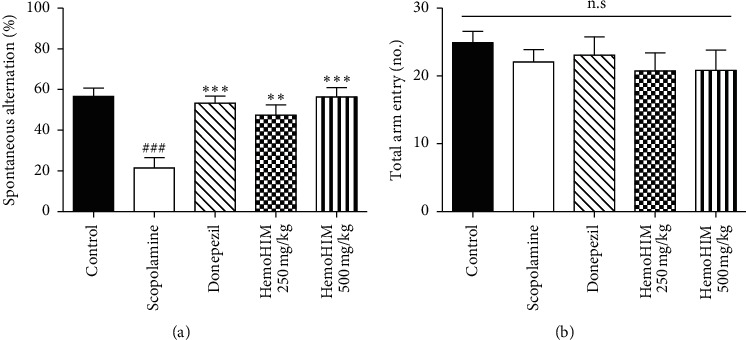
The effects of HemoHIM on scopolamine-induced memory impairment in the Y-maze test. Spontaneous alternation (%) was calculated as follows. % = (Number of alternations/Total arm entries − 2) × 100. Spontaneous alternation (a) and numbers of total arm entry (b) were measured for Y-maze test. Ninety minutes before Y-maze, mice were treated with HemoHIM (250 and 500 mg/kg, p.o.) or donepezil (1 mg/kg, p.o.) as a positive control. Memory impairment was induced by scopolamine treatment (1 mg/kg, i.p.). Thirteen different animals were used per group. The data are expressed as the mean ± S.E.M. Significant difference from control group (###*P* *<* 0.001) and from scopolamine-treated group (^*∗∗∗*^*P* *<* 0.001, ^*∗∗*^*P* *<* 0.01).

The Y-maze test was performed to assess whether HemoHIM improved memory impairment induced by scopolamine. As shown in Figure 2(a), spontaneous alternation in the scopolamine-treated group was significantly decreased compared with the control group (21.50 ± 5.07% and 56.67 ± 4.01%, respectively) (*P* < 0.05). Furthermore, HemoHIM significantly reversed the reduced alternation induced by scopolamine at 250 mg (47.38 ± 5.11%) and 500 mg/kg (56.41 ± 4.57%). In addition, the effects of HemoHIM on spontaneous alternation were similar to that of donepezil (53.27 ± 3.56%). However, the number of total arm entry had not significantly changed compared with each experimental group (Figure 2(b)). Therefore, it was confirmed that HemoHIM or donepezil did not affect mice locomotor activity.

**Figure 3:**
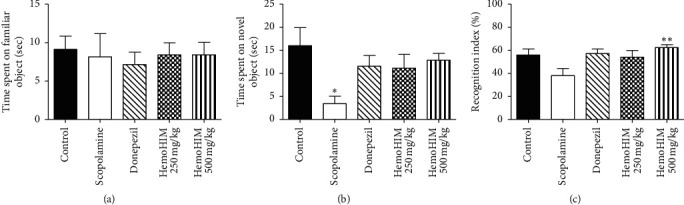
The effects of HemoHIM on scopolamine-induced memory impairment in the NORT. (a) Time spent on familiar object (sec), (b) time spent novel object (sec), and (c) Recognition index (%). Ninety minutes before Y-maze, mice were treated with HemoHIM (250 and 500 mg/kg, p.o.) or donepezil (1 mg/kg, p.o.) as a positive control. Memory impairment was induced by scopolamine treatment (1 mg/kg, i.p.). Thirteen different animals were used per group. The data are expressed as the mean ± S.E.M. Significant difference from control group (#*P* < 0.05) and from scopolamine-treated group (^*∗∗*^*P* < 0.01).

The NORT is a commonly used behavioral test for the investigation of various aspects of learning and memory in rodents. Mice in the scopolamine-treated group spent significantly longer time exploring the familiar object than exploring the novel object (Figure 3(b)). The percentage recognition index was not increased in the positive control and HemoHIM 250 mg/kg group (57.17 ± 4.01% and 54.01 ± 5.83%, respectively). However, the recognition index in the HemoHIM 500 mg/kg group was notably higher than that in the scopolamine-treated group (62.65 ± 2.37% and 38.02 ± 6.15%, respectively, Figure 3(c)). Mice in the HemoHIM 500 mg/kg group spent more time exploring the novel object than exploring the familiar object. Therefore, the high concentration of HemoHIM was confirmed to improve cognitive impairment.

**Figure 4:**
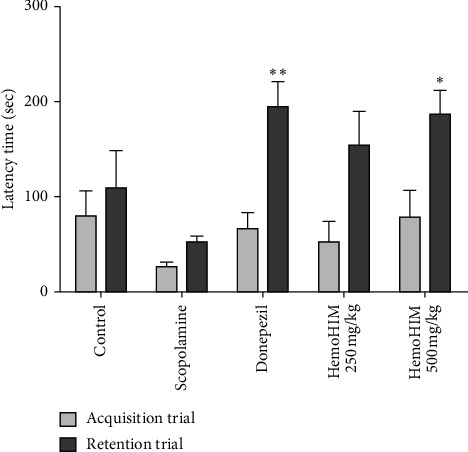
Effects of HemoHIM (250 and 500 mg/kg, p.o.) on scopolamine-induced memory impairment in the passive avoidance test in mice. The results from the acquisition trial and retention trial were presented. Ninety minutes before Y-maze, mice were treated with HemoHIM (250 and 500 mg/kg, p.o.) or donepezil (1 mg/kg, p.o.) as a positive control. Memory impairment was induced by scopolamine treatment (1 mg/kg, i.p.). Thirteen different animals were used per group. The data are expressed as the mean ± S.E.M. Significant difference from scopolamine-treated group (^*∗∗*^*P* < 0.01, ^*∗*^*P* < 0.05).

The effects of HemoHIM on long-term memory were investigated using the PAT (Figure 4). The results showed the time delay to enter into the dark room was significantly reduced in the scopolamine-treated group compared with the control group (111.07 ± 59.81 sec and 61.6 ± 7.96 sec, respectively, Figure 4). Conversely, donepezil (162.06 ± 31.19 sec) and HemoHIM (500 mg/kg) significantly increased the time latency (171.10 ± 30.44 sec *P* < 0.01 and *P* < 0.05, respectively).

### 3.2. Effects of HemoHIM on Cholinergic Activity in the Hippocampus

**Figure 5:**
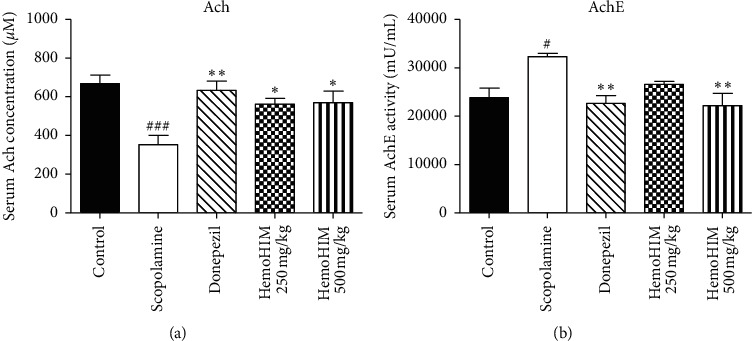
The effects of HemoHIM on (a) Ach concentration and (b) AchE activity in the hippocampus. Ach levels (*n* = five per group) and AchE (*n* = 6 per group) in the hippocampus were determined using the ELISA kit. The data are expressed as the mean ± S.E.M. Significant difference from control group (###*P* < 0.001) and from scopolamine-treated group (^*∗∗∗*^*P* < 0.001, ^*∗*^*P* < 0.05).

In previous studies, several natural compounds or extracts with AchE inhibitory activity were reported to exhibit significant cognitive improvement effects. Therefore, hippocampal Ach and AchE activities were measured in the present study to determine the fundamental mechanism underlying the possible alteration of Ach and AchE levels. The activity of Ach slightly decreased in the scopolamine-treated group (2.61 ± 0.25) compared with controls (3.05 ± 0.06). However, donepezil and HemoHIM 500 mg/kg groups showed a significant increase in Ach levels (3.39 ± 0.05 and 3.11 ± 0.11, respectively, Figure 5(a)). As shown in Figure 5(b), the scopolamine-increased activity of AchE was significantly reduced by the administration of HemoHIM (500 mg/kg) and by the donepezil treatment (*P* < 0. 05 and *P* < 0.001, respectively).

### 3.3. Effects of HemoHIM on the mRNA Levels of Ach-Related Metabolic Enzymes and Receptors

**Figure 6:**
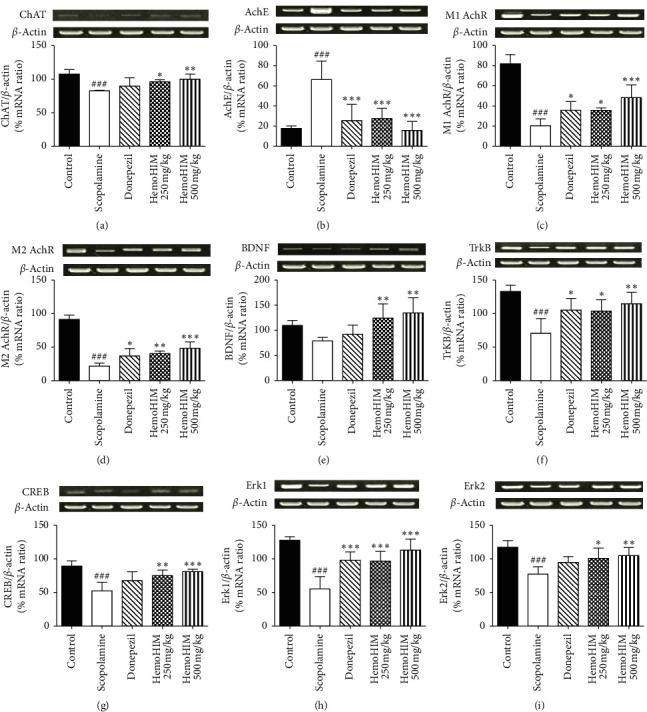
The effects of HemoHIM on mRNA expression of (a) ChAT, (b) AchE, (c) mAchR M1, (d) mAchR M2, (e) BDNF, (f) TrkB, (g) CREB, (h) Erk1, and (i) Erk2 in the scopolamine-induced model. The mRNA expression was measured by RT-PCR using specific primers. *β*-actin levels were compared for the equal loading control. Quantitative analysis of relative expression for BDNF/*β*-actin was represented as graph. The data are expressed as the mean ± S.E.M. (*n* = 4∼5 per group). Significant difference from control group (###*P* < 0.001) and from scopolamine-treated group (^*∗∗∗*^*P* < 0.001, ^*∗∗*^*P* < 0.01, ^*∗*^*P* < 0.05).

The effects of HemoHIM on cognitive memory enhancement were investigated. The Ach-related metabolic enzymes and receptors were analyzed using RT-PCR. As shown in Figure 6(a), the ChAT activity decreased more in the scopolamine-treated group than in controls; however, the HemoHIM 500 mg/kg group showed significantly increased ChAT activity compared with the scopolamine-treated group. The mAchR (M1 and M2) mRNA expression was significantly lower in the scopolamine-treated group than in controls (Figures 6(c), 6(d)). However, HemoHIM strongly increased M1 and M2 expression in a dose-dependent manner. Scopolamine administration notably upregulated AchE expression in the hippocampus. Administration of HemoHIM and donepezil induced a significant attenuation of AchE expression (Figure 6(b)). Scopolamine treatment not only reduced BDNF but also decreased the amount of ERK and CREB. Conversely, the HemoHIM groups showed significantly increased ERK, BDNF, and CREB expressions (Figures 6(e), 6(g)–6(i)).

### 3.4. Effects of HemoHIM on the Expression of BDNF Activation

**Figure 7:**
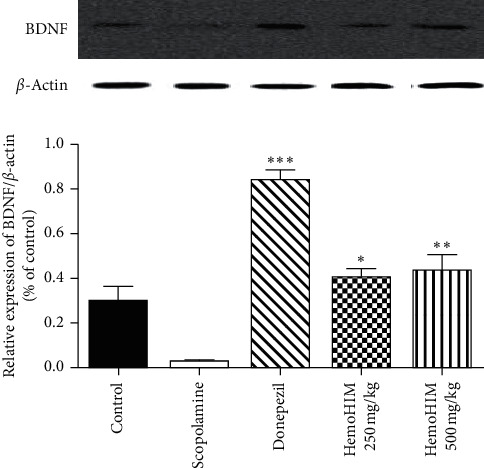
The effects of HemoHIM on BDNF protein levels in the hippocampus. Protein expression was measured by western blot analysis using anti-BDNF-specific antibody. *β*-actin levels were compared for the confirmation of an equal amount of protein loading in each group. Quantitative analysis of relative expression for BDNF/*β*-actin was represented as graph. The data are expressed as the mean ± S.E.M. ^*∗∗∗*^*P* < 0.001, ^*∗∗*^*P* < 0.01, and ^*∗*^*P* < 0.05 compared with the scopolamine-treated group.

To examine whether HemoHIM influences protein expression associated with long-term memory, western blotting for BDNF in hippocampal tissue was performed (Figure 7). Treatment with HemoHIM (500 mg/kg) significantly upregulated BDNF expression in the hippocampus. The positive control also showed significantly increased BDNF expression. The result indicates HemoHIM may protect against scopolamine-induced amnesia *via* mechanisms associated with neuronal cell activation.

## 4. Discussion

The herbal preparation HemoHIM is a functional health food, which has obtained certification by the Ministry of Food and Drug Safety (MFDS) to improve immune function. HemoHIM consists of hot water extract with a polysaccharide fraction of herbs, *Angelica gigas* Nakai (Apiaceae), *Cnidium officinale* Makino (Umbelliferae), and *Paeonia japonica* Miyabe (Paeoniaceae). Various effects of immune enhancement in HemoHIM such as immune cell activation, immune hematopoietic recovery, tissue regeneration, and antioxidant effects have been reported [7, 9, 11, 12]. The major components of HemoHIM are gallic acid, chlorogenic acid, paeoniflorin, and nodakenin. In previous studies, Kim et al. and Kwon et al. reported that nodakenin and chlorogenic acid might be useful for the treatment of cognitive impairment *via* the antioxidant activity and the enhancement of cholinergic signaling [18, 21].

In the present study, the memory-enhancing effects of HemoHIM were investigated in a scopolamine-induced mouse model of amnesia using behavioral tests and the related mechanisms. Scopolamine, a muscarinic antagonist, can cause learning and memory deficits by disrupting cholinergic activity. Consequently, scopolamine has often been widely used to screen potential agents as effective treatment for antiamnesia or dementia in experimental murine models [18, 27, 31, 32]. In the present investigation, the learning and memory function were evaluated in experimental animals using the Y-maze, NORT, and PAT. These behavioral tests are useful for studying short- and long-term memory by manipulating the retention interval [30, 33]. In previous studies, scopolamine-treated animals showed a lower discrimination index in the NORT, a higher transfer latency in the elevated plus-maze test, and a longer latency in the Morris water maze test [34, 35]. Increasing spontaneous alternation indicates that learning and memory have been restored. In the Y-maze task, HemoHIM administration (250 or 500 mg/kg) significantly increased spontaneous alternation behavior in mice, ameliorating the decreased alternation behavior that was induced by scopolamine. Furthermore, this was similar to the effects of donepezil. The NORT is universally recognized for short- or long-term memory. In the present study, scopolamine-treated mice exhibited significantly decreased exploration time showing a novelty preference, indicating that scopolamine causes impairment of object recognition memory. In contrast, mice administered HemoHIM (500 mg/kg) showed novelty preference by spending more time on the novel object relative to the familiar object. The PAT is a fear-motivation test that is classically used to assess short- or long-term memory using the natural tendency of rodents for dark places [36]. The amount of time a mouse stays in the brightly lit box before entering the dark box, where an electric shock is administered, is considered the latency time. Longer latency time indicates improvement of learning and memory function. In the present study, latency time was compared and difference was not observed between groups in the acquisition trial. However, the latency time was reduced in the scopolamine-treated group in the retention trial. The results showed that HemoHIM treatment improved latency time during trials, indicating the amelioration of learning impairment.

Dementia, which appears to be the cause of aging and Alzheimer's disease, is a neurodegenerative brain disease and considered an important social problem as well as a major disease in modern society [37]. Memory impairment is the earliest and the most prominent symptom of dementia. Memory is related to the cerebral cortex, especially the hippocampus. The cholinergic deficits are neuropathological occurrences that are consistently associated with memory impairment in the hippocampus and play an important role in memory formation and cognition [38, 39]. In addition, reduction in memory function is correlated with dementia. In several reports, choline uptake and Ach synthesis were reduced in patients with dementia [40, 41]. In addition, many approaches lead to enhancing the concentration of acetylcholine. Many studies have been conducted to increase the content of endogenous Ach by blocking the action of AchE, which hydrolyzes Ach. As in previous studies, the administration of scopolamine reduced Ach concentration and elevated AchE activity in the present study [27]. On the other hand, HemoHIM was able to confirm Ach significant increase and AchE activity inhibition in the serum and hippocampus (Figures 3, 4). Conversely, HemoHIM significantly increased Ach and inhibited AchE activity in the serum and hippocampus (Figure 3, 4). HemoHIM increased the Ach level similar to donepezil, which was used as a control drug. These findings indicate that HemoHIM decreases the activity of AchE, which hydrolyzes Ach, consequently increasing the endogenous Ach and improving cholinergic neurotransmission.

To investigate the effects of HemoHIM, the expression of receptors that can influence the synthesis, release, and degradation of Ach, a neurotransmitter that plays a central role in memory and learning, was investigated. ChAT, a transferase enzyme responsible for the synthesis of the Ach, is important for performing basic brain functions. mAchRs are Ach receptors that form G protein-coupled receptor complexes in the cell membranes. mAchRs M1 and M2 are the most abundant muscarinic receptors in the hippocampus and were shown to exert cognitive effects [42], and M2-deficient mice had disrupted hippocampal Ach homeostasis [43]. Decreased ChAT and mAchR activity, especially in the hippocampus, has been previously reported in patients with dementia [44]. In the present study, reduced ChAT and mAchRs M1 and M2 levels were observed in the scopolamine-treated group; however, the expression levels were increased in the HemoHIM 500 mg/kg group. Thus, based on the findings from the present and previous studies, HemoHIM likely improves Ach synthesis by increasing ChAT and mAchRs M1 M2 levels and decreasing AchE activity. In addition, expression of transcription and neurotrophic factors such as CREB and BDNF was observed. BDNF binds to its receptor TrkB and activates tyrosine kinase, a trigger for various intercellular signaling pathways [45]. The BDNF and TrkB expression was significantly increased in the HemoHIM groups. These neurotrophic factors are being developed as biomarkers and new drug targets for mental nervous system diseases such as memory impairment and depression and/or anxiety. CREB is one of the key signaling molecules involved in learning and retrieval of fear-based long-term memory and are activated by phosphorylation on serine 133, which can be controlled by ERK signaling [46]. Especially, BDNF is a member of the neurotrophin family of growth factors and appears essential for molecular mechanisms of synaptic transmission and synapse plasticity in the central nervous system [47]. Our result is HemoHIM (500 mg/kg) treatment significantly upregulated BDNF expression in the hippocampus. Therefore, HemoHIM high dose can increase BDNF expression and help improve cognitive memory. However, further studies are needed to identify the molecular biological mechanism of action and signal pathways other than BDNF.

## 5. Conclusion

The present study was conducted to evaluate the antiamnesic activity of HemoHIM in a scopolamine-induced memory impairment murine model. HemoHIM administration effectively ameliorated cognitive deficits determined using PAT, NORT, and Y-maze behavioral tests. HemoHIM effectively inhibited AchE activity. Furthermore, HemoHIM may attenuate memory impairment by activating the BDNF-ERK-CREB pathway. These results indicate that HemoHIM can be used pharmaceutically for the treatment or prevention of amnesia and dementia and can be used as a supplement to improve dementia symptoms.

## Figures and Tables

**Table 1:** Primer sequences used for reverse transcriptase PCR.

Gene	Direction	Sequence (5′ to 3′)
AchE	Forward	AGAAAATATTGCAGCCTTTG
	Reverse	CTGCAGGTCTTGAAAATCTC
ChAT	Forward	AGGGTGATCTGTTCACTCAG
	Reverse	TCTTGTTGCCTGTCATCATA
M1 AchR	Forward	CAGAAGTGGAGATGCC
	Reverse	GAGCTTTTGGGAGGCTGCTT
M2 AchR	Forward	TGCTGTGGCCTCCAATATGA
	Reverse	TGACCCGACGACCCAACT
BDNF	Forward	AGCTGAGCGTGTGTGACAGTAT
	Reverse	CCGAACATACGATTGGGTAGTT
Trk *β*	Forward	GCACATCGCTCAGCAAATCG
	Reverse	ACAACTCCCAGGCTCCAGAC
CREB	Forward	CCCAGGGAGGAGCAATACAG
	Reverse	GGGAGGACGCCATAACAACT
ERK1	Forward	ACCGTGACCTCAAGCCTTCC
	Reverse	GATGCAGCCCACAGACCAAA
ERK2	Forward	TTGCTGAAGCACCATTCAAG
	Reverse	ACGGCTCAAAGGAGTCAAGA
*β*-actin	Forward	GCCATGTACGTAGCCATCCA
	Reverse	GAACCGCTCATTGCCGATAG

## Data Availability

The data used to support the findings of this study are available from the corresponding author upon request.
